# The Importance of Body Part Labeling to Enable Enterprise Imaging: A HIMSS-SIIM Enterprise Imaging Community Collaborative White Paper

**DOI:** 10.1007/s10278-020-00415-0

**Published:** 2021-01-22

**Authors:** Alexander J. Towbin, Christopher J. Roth, Cheryl A. Petersilge, Kimberley Garriott, Kenneth A. Buckwalter, David A. Clunie

**Affiliations:** 1 Department of Radiology, Cincinnati Children’s Hospital, University of Cincinnati College of Medicine, Cincinnati, OH USA; 2 Department of Radiology, Duke University Medical Center, Durham, NC USA; 3 Department of Radiology, University of Pittsburgh Medical Center, Pittsburgh, PA USA; 4 NetApp Global Healthcare, CA Sunnyvale, USA; 5 Department of Radiology, University of Michigan School of Medicine, Ann Arbor, MI USA; 6 Pixelmed Publishing, LLC, Bangor, PA USA

**Keywords:** Enterprise Imaging, DICOM, Standards, Medical Photography, Ontology, Photo documentation

## Abstract

In order for enterprise imaging to be successful across a multitude of specialties, systems, and sites, standards are essential to categorize and classify imaging data. The HIMSS-SIIM Enterprise Imaging Community believes that the Digital Imaging Communications in Medicine (DICOM) *Anatomic Region Sequence*, or its equivalent in other data standards, is a vital data element for this role, when populated with standard coded values. We believe that labeling images with standard Anatomic Region Sequence codes will enhance the user’s ability to consume data, facilitate interoperability, and allow greater control of privacy. Image consumption—when a user views a patient’s images, he or she often wants to see relevant comparison images of the same lesion or anatomic region for the same patient automatically presented. Relevant comparison images may have been acquired from a variety of modalities and specialties. The *Anatomic Region Sequence* data element provides a basis to allow for efficient comparison in both instances. Interoperability—as patients move between health care systems, it is important to minimize friction for data transfer. Health care providers and facilities need to be able to consume and review the increasingly large and complex volume of data efficiently. The use of *Anatomic Region Sequence*, or its equivalent, populated with standard values enables seamless interoperability of imaging data regardless of whether images are used within a site or across different sites and systems. Privacy—as more visible light photographs are integrated into electronic systems, it becomes apparent that some images may need to be sequestered. Although additional work is needed to protect sensitive images, standard coded values in *Anatomic Region Sequence* support the identification of potentially sensitive images, enable facilities to create access control policies, and can be used as an interim surrogate for more sophisticated rule-based or attribute-based access control mechanisms. To satisfy such use cases, the HIMSS-SIIM Enterprise Imaging Community encourages the use of a pre-existing body part ontology. Through this white paper, we will identify potential challenges in employing this standard and provide potential solutions for these challenges.

## Introduction

Enterprise imaging is at an early crossroads. The ability to share medical images meaningfully between entities, or even internally within an entity, is dependent on a common language. Today, we rely on “translators”, such as data value and code “crosswalks”, to establish a common understanding of the information from disparate sources. This approach treats a symptom—the need to translate data from one entity to another—but it does not address the root cause, which is the failure to deploy a universal medical terminology.

For enterprise imaging to grow in an organized manner, we believe that standard metadata elements populated with standard coded values are needed to categorize and classify imaging data. Despite the existence of mature data language and communication standards, such as LOINC, SNOMED CT, DICOM, HL7 version 2, as well as new standards like FHIR, there is limited awareness of these standards among providers at the point of data creation [1–6]. Mature acquisition and storage systems may automatically apply the appropriate standards, but less mature devices and applications may rely on the end-user to assign metadata data elements to newly created data. This process is error-prone and inefficient. Many specialties, new to digital imaging and not experienced with large-scale, high-throughput image acquisition and storage, will be challenged to apply metadata to their images.

The emergence of smartphones and affordable digital cameras has led to an explosion in the number of images obtained during routine patient encounters. Because images are easy to acquire at the point of care and have become a valued asset in the medical record, imaging data has become one of the fastest-growing segments of acquired medical information. It is estimated that over 90% of healthcare data is comprised of clinical images and related metadata [7, 8]. Unfortunately, like personal photographs captured on our smartphones, such medical images may be stored with only sparse relevant contextual information and content metadata.

As practitioners in the field of enterprise imaging, which impacts nearly every clinical service line, we recognize a need for the use of standardized medical terminology for procedures, anatomy, acquisition method, and related concepts [9, 10]. Such standardization will enable interoperability, enhance care coordination, security, patient safety and privacy, advanced analytics, and artificial intelligence.

Since this is such a broad topic, we suggest that a concerted effort be made to select an appropriate standard data element and coded terminology for the anatomy included in an image. An illustrative example will show why anatomy is an important data element. We will identify several challenges that need to be solved for a single standard data element to be used across all medical use cases.

In this white paper, we will identify the Digital Imaging and Communications in Medicine (DICOM) data elements that we believe to be the most relevant for data consumption, interoperability, and data security [5]. At the first mention of a DICOM data element in the text, the hexadecimal numeric data element tag will be displayed in parenthesis following the italicized DICOM field name, as is the standard convention. We have chosen to use the DICOM standard for this manuscript based on its stability and its widespread adoption across multiple specialties within hospitals and clinics across the world [13–21].

We recognize that not all enterprise imaging solutions use DICOM for visible light imaging applications or encapsulate consumer image files (such as JPEG images) in DICOM; however, it is clear that DICOM defines a standard solution to the problem described. We believe that the concepts elucidated in this manuscript, and in particular the use of standard codes, can be translated to other means of associating metadata with images. Other standard mechanisms may be used for the exchange of images, such as Integrating the Healthcare Enterprise (IHE) Cross-Enterprise Document Sharing (XDS.b) [11] and Cross-enterprise Document Sharing for Imaging (XDS-I.b) [12]. XDS-I.b defines a mechanism for using DICOM Anatomic Region coded information in the XDS eventCodeList metadata. Similarly, the same codes can be used in data elements within the proprietary database of an electronic health record (EHR). Though it is theoretically possible to contrive a means to insert anatomic information into photographic images, such as in an EXIF marker segment embedded in JPEG images, there is no standard EXIF or JPEG field defined for this.

## Framing Use Case

The following use case will be referred to throughout this white paper. While this case is lengthy, we believe that it describes a typical scenario related to a single diagnosis. Additionally, we believe that this use case highlights the complexity of enterprise imaging and the urgency of a solution to enable providers to use imaging information efficiently and safely.

Consider a patient with a right-sided nasopharyngeal mass. She initially presents to her family medicine physician who examines the mass and takes a photograph using a personal smartphone. Based on the patient’s history of Epstein-Barr virus and allergic rhinitis, the physician’s differential diagnosis includes nasal polyps and nasopharyngeal carcinoma. Because of the differential diagnosis, the physician refers the patient to an otolaryngologist for further evaluation. The patient’s appointment is scheduled for 3 weeks hence. In the interim, the mass enlarges and begins to bleed. Concerned, the patient takes a picture herself with her smartphone and emails it to her doctor.

The family medicine physician orders an outpatient MRI to evaluate the mass. A neuroradiologist makes a presumptive diagnosis of nasopharyngeal carcinoma. After waiting for 3 weeks, the patient sees the otolaryngologist in a private, office-based practice. This otolaryngologist is also concerned that the patient has a nasopharyngeal carcinoma and performs office nasolaryngoscopy. Several pictures are acquired, and a biopsy performed. The patient is told to expect minimal bleeding and to call the office if she is concerned.

That evening, the packing material becomes soaked with blood and falls out of the nasal cavity. The patient takes a photograph of the packing with her smartphone and emails it to her otolaryngologist. The otolaryngologist reviews the image and recommends that the patient goes to the emergency department of a local hospital for further evaluation.

In the emergency department, the nurse uses a shared departmental smart device to take a photograph of the nasal cavity and shows it to the emergency department physician. After review, the emergency department physician requests that the photo be sent to the on-call otolaryngologist (from a different, hospital-based practice than the first otolaryngologist) and asks the consulting otolaryngologist to see the patient emergently. Once in the emergency room, the on-call otolaryngologist performs another nasolaryngoscopic examination, takes photographs, then cauterizes a bleeding vessel. The patient is discharged to recuperate.

Meanwhile, the specimen from the earlier biopsy is sent to the pathology department where the pathologist obtains gross specimen photographs using a digital single-lens reflex (dSLR) camera and submits the tissue for fixing, embedding, and staining followed by digital whole slide scanning. The pathologist evaluates the tissue using a virtual microscopy application and signs out the case as nasopharyngeal carcinoma.

With the malignant diagnosis confirmed, the original otolaryngologist takes over the care of the patient and orders a chest CT and a whole-body PET/CT to evaluate the disease extent. Imaging is performed at a different outpatient imaging facility than the original MRI. The nuclear medicine physician reports regional lymph node involvement. The otolaryngologist refers the patient to an oncologist to coordinate further care.

The oncologist recommends that the patient undergo radiation therapy and chemotherapy and refers her to a radiation oncologist. The radiation oncologist obtains CT images for initial treatment planning and at subsequent visits. At predefined intervals, repeat PET/CT and MRI are also performed to monitor the patient’s response to therapy. These follow-up scans reveal residual tumor in the neck lymph nodes.

The otolaryngologist is informed of the findings and performs a radical lymph node dissection. During surgery, multiple photographs and brief videos are recorded using a dSLR camera. The lymph node specimens are sent for histopathology, and gross photographs and whole slide scans are acquired. Meanwhile, the patient is seen in the wound care clinic several times to ensure that the neck incision healed completely after the extensive surgery and radiation therapy. Photographic documentation is performed during each visit to monitor healing.

All the images in this scenario were obtained over 6 months. During that time, the patient received medical attention for other chronic medical conditions as well as routine screening studies such as mammography and dermatologic mole mapping. After the lymph node resection, the patient noticed swelling in her right breast. When she mentioned the swelling at the screening mammography visit, the technologist took a photograph of the swelling along with the digital mammograms and converted the study to a diagnostic mammogram. A digital breast tomosynthesis study was obtained and the breast imager performed a targeted ultrasound.

During the scenario, at least 23 imaging studies were performed by eight specialty services. The imaging studies and the facilities, specialties, and departments performing the imaging study are highlighted in Table 1.

**Table 1:** Table highlighting the different imaging studies for the patient in the framing use case. For each imaging study, the following DICOM data elements are populated: modality, study description, institutional department type code sequence, anatomic region sequence, and primary anatomic structure sequence. For each element, current potential DICOM values are populated

Image acquisition point	Workflow step	Modality (0008, 0060)	Study Description (0008,1030)^a^	Institutional Department Type Code Sequence (0008,1041)^c^	Anatomic Region Sequence (0018,0015)	Image Laterality (0020,0062)	Primary Anatomic Structure Sequence (0008,2228)^e^
1	Family medicine physician-acquired photograph of nasal mass	XC	Photo-Head and Neck^b^	Primary Care Department	Head and Neck		Nasopharynx
2	Patient-acquired photograph of nasal mass	XC	Photo-Head and Neck^b^		Head and Neck		Nasopharynx
3	MRI	MR	MR Neck w and wo IV Contrast	Radiology	Neck		
4	Nasolaryngoscope photograph	ES	Nasolaryngoscope^b^	Otorhinolaryngology	Nasopharynx		
5	Patient-acquired photograph of packing material	XC	Photo-Head and Neck^b^		Head and Neck		
6	Nurse-acquired photograph of bleeding nasal mass	XC	Photo-Head and Neck^b^	Accident and Emergency	Head and Neck		Nasopharynx
7	Nasolaryngoscope photograph	ES	Nasolaryngoscope^b^	Otorhinolaryngology	Nasopharynx		Pharyngeal recess
8	Gross specimen-intake workflow	XC or GM	Gross Specimen—Head and Neck^b^	Pathology	Nasopharynx		Pharyngeal recess
9	Pathology slide scanning workflow	SM	Whole Slide-Head and Neck^b^	Histopathology			Pharyngeal recess
10	CT	CT	CT Chest wo IV Contrast	Radiology	Chest		
11	PET/CT	PT, CT	PT/CT Tumor Localization Whole Body	Nuclear Medicine	Whole Body		
12	Radiation Oncology planning CT	RTPLAN	CT Treatment Planning Complex	Radiotherapy	Head and Neck		
13	PET/CT	PT, CT	PT/CT Tumor Localization Whole Body	Radiology	Whole Body		
14	MRI	MR	MR Neck w and wo IV Contrast	Neuroradiology	Neck		
15	Surgical photos and videos from lymph node dissection	XC	Photo-Surgical-Head and Neck^b^	Otorhinolaryngology	Neck		Upper jugular lymph node
16	Gross specimen-intake workflow	XC or GM	Gross Specimen-Head and Neck^b^	Pathology	Neck		Upper jugular lymph node
17	Pathology slide scanning workflow	SM	Whole slide-Head and Neck^b^	Histopathology			Upper jugular lymph node
18	Nurse-acquired wound care photographs	XC	Photo-Wound-Head and Neck^b^	Wound Care^d^	Neck	R	
19	Mammography	MG	Mammo Breast Screening	Radiology	Breast	B	
20	Medical photographer-acquired dermatology photographs	XC	Photo-Mole Map^b^	Dermatology	Whole Body		
21	Radiology technologist-acquired photograph	XC	Photo-Breast^b^	Radiology	Breast	R	
22	Tomosynthesis	MG	Mammo Breast Diagnostic Tomosynthesis Bilateral	Radiology	Breast	B	
23	Ultrasound	US	US Breast Right	Radiology	Breast	R	

^a^The study description represents is a text element. LOINC-RSNA Radiology playbook terms are used to populate the study description element for radiologic studies [33]

^b^No LOINC-RADLEX playbook exists for this type of imaging study. A sample procedure description is thus included

^c^The specialty Institutional Department Type Code Sequence is presented. The data element has been left blank for patient-acquired photographs [38]

^d^No Institutional Department Type Code Sequence exists for the specialty. A sample specialty name is used

^e^A sample anatomic structure has been selected to fill the Primary Anatomic Structure Sequence. This element is left blank when large field of view imaging is performed

### Clinical Overview

Generally, providers working in the EHR need access to relevant imaging at the point of care. To support efficient workflow, providers expect imaging and the associated notes and reports to be returned to them expeditiously, to be visible in-line with their clinical care workflow, and ideally to be made available automatically [10–12]. Different specialty providers, however, prioritize different imaging needs. For example, our patient’s mole mapping and breast photography do not need to be presented automatically to the otolaryngologists. However, a sequence of relevant head and neck CT, PET, and MR studies and clinical photographs would be helpful to track the nasopharyngeal lesion over time and assess the patient’s response to therapy. Similarly, the radiologist interpreting the diagnostic mammogram for breast swelling needs to see prior breast imaging as well as the patient’s whole-body PET/CT studies, but may not be concerned about the diagnostic or photographic head and neck imaging.

Specialists who have performed and interpreted diagnostic imaging for many years are aware that automating image presentation workflows requires metadata data elements [10, 13–15]. These metadata elements can be used to create “display protocols.” Default display protocols are “the series of actions performed to arrange images for optimal viewing [12, 16].” They are used in medical imaging to prescribe how images of different types are organized with respect to one another, and how a current imaging study is displayed along with its relevant comparison studies. The relevant comparison studies vary depending on the type of imaging study that is opened.

Different users may have different pre-configured preferences. For example, one otolaryngologist may define the relevant comparison for a nasolaryngoscopic examination as the previous examination only, while a second otolaryngologist may define the relevant comparison studies as being those that include any radiology, pathology, or otolaryngologic images of the head and neck region.

## Problems to Be Solved

### Problem: Image Consumption

An expectation of enterprise imaging is that it will enable more coordinated care when providers have greater access to images obtained throughout their patient’s medical journey as opposed to having images sequestered in departmental siloes, or not stored at all. Although we believe this to be true, success is predicated on the concept that the images not only be accessible, but relevant and intelligently integrated into the workflow. Specifically, EHR and imaging software needs to automatically present providers with the images most relevant to their patient’s current health care complaint. This presentation of images is achieved by using associated metadata to search automatically and identify appropriate multimedia objects. Several data elements can be used to determine relevance. Some, such as study date, the performing or ordering provider, and the imaging department, can be attached automatically through the order-based or encounter-based image capture workflow [11]. Other fields such as anatomy and procedure description may need to be captured at the time of imaging, especially if they are not implicit in the order or when there is no order.

After consideration of the different potential DICOM data element data fields, we believe that the *Anatomic Region Sequence* (0008,2218) and the *Institutional Department Name* (0008,1040) are the two key data elements with the greatest potential to help end-users identify relevant associated imaging studies. In our use case, images were obtained at multiple time points, at multiple locations, by multiple providers from multiple clinical specialties using multiple imaging modalities. Currently, a provider would have difficulty if he or she were trying to find all of the images that related specifically to the framing patient’s nasopharyngeal carcinoma.

Throughout the next section, we will explore several potential metadata elements that can be used to identify images for efficient consumption. In this assessment, we will describe the element, its benefits, and, if applicable, why it may be deficient to adequately address the end-user’s need for image consumption, interoperability, and privacy or security. Key recommendations are summarized in Table 2.

**Table 2:** Summary of key recommendations

Recommendations related to body parts terminology
1. *Anatomic Region Sequence *is the most useful single DICOM data element to drive image consumption across specialties
2. The *Anatomic Region Sequence *should be populated using a standard body part ontology
3. Providers and industry representatives should work together to identify and select an existing body part ontology that will meet the varied needs of specialists and the general end-users across the enterprise
4. The selected standard body part terminology should be an ontology including anatomic relationships, so that concepts with a higher degree of specificity are relatable to those with a lower degree of specificity

Other recommendations
1. Information encoded in plain text fields, such as* Study Description* and *Series Description*, is not standardized and should not be relied upon to drive workflow or system behavior
2. *Institutional Department Type Code Sequence *should be populated with an appropriate code based on the department or division performing the imaging study
3. *Reason For Performed Procedure Code Sequence *and *Admitting Diagnoses Code Sequence *should be populated, if the appropriate information is available. However, it is recognized that this data is unlikely to be updated and thus will become outdated as the knowledge of a patient’s underlying illness grows

### Anatomic Region Sequence (0008,2218), Primary Anatomic Structure Sequence (0008,2228), and Body Part Examined (0018,0015)

Those involved in diagnostic radiology recognized that the integration of metadata from information systems is critical to the success of digital imaging. The development and adoption of standard protocols, message, and image formats support this integration [17, 18]. The original American College of Radiology (ACR)-National Electrical Manufacturers Association (NEMA) standard defined many useful data elements describing images including anatomical positioning but did not explicitly describe the anatomy [19]. The first release of the DICOM standard provided a short list of capitalized string values to use in a new data element, *Body Part Examined* (0018,0015), for Computed Radiography (CR), and a much longer list for the now-retired element, *Anatomic Structure* (0008,2208), for ultrasound [20]. Formal use of externally defined standard terminologies rather than home-grown string codes was not introduced until several years later and is now well established [21–23].

DICOM provides data elements for identifying the general region of the patient anatomy examined using coded terminology as well as the principal structures within that region that are the target of a particular image [24]. The coded data element that DICOM provides to describe the general region of the body being examined is *Anatomic Region Sequence*. This data element is available for use in all types of DICOM images [25]. Sets of codes that may be used to populate this data element are also provided in the standard. Most of them are selected from SNOMED CT [26–28]. The *Anatomic Region Sequence* is the data element intended to replace the text field, *Body Part Examined*. The current recommendation in DICOM is to avoid the use of *Body Part Examined*.

When there is a need to describe a more specific anatomic location in addition to the region, one or more codes can be sent in the image level element, *Primary Anatomic Structure Sequence* (0008,2228). For example, a specific anatomic structure of interest within the image, such as a lymph node within the neck, can be described this way.

Both *Anatomic Region Sequence* and *Primary Anatomic Structure Sequence* codes may have a list of modifiers, to describe a sub-region (e.g., medial, lateral, superior, inferior, lobe, quadrant), sub-location (e.g., subcapsular, peripheral, central), or configuration (e.g., distended, contracted). Laterality is better separately encoded, rather than encoded in modifiers. However, both practices exist.

*Anatomic Region Sequence*, by design, allows for only a single code to be present. When an imaging study spans multiple regions, traditionally each region is named separately. For example, head and neck or chest, abdomen, and pelvis are single pre-coordinated codes provided for the combined body parts. Currently, the pre-coordinated codes are mostly derived from SNOMED CT. If a body part location does not exist in SNOMED CT, it is provided from the Foundational Model of Anatomy.

In our nasopharyngeal carcinoma clinical use case, most imaging studies would be identified using the same *Anatomic Region Sequence* data element value of *Head and Neck*. Perhaps, the only relevant imaging studies for that use case that would not be identified using the *Anatomic Region Sequence* are the CT of the chest and the whole-body PET/CT. We will discuss the special example of the photograph of the dislodged packing material later in this paper.

Although we believe that *Anatomic Region Sequence* will prove to be the most useful single data element to group relevant comparative imaging studies, there will be instances where this data element will be incomplete. For example, a patient with systemic lupus erythematosus may have a rash of the face, arthritis of the hands, and interstitial lung disease. In this example, no data element would identify all the imaging studies related to the single systemic disease process, which involves multiple organs and multiple body parts. Such sophisticated questions require a more profound source of knowledge to resolve, such as an ontology that recognizes which organ systems and anatomical locations may be involved in a disease process, and a reasoner designed to perform such queries. The value of using a standard source of anatomical concepts and encoding them in *Anatomic Region Sequence* is a prerequisite for such approaches but is not sufficient alone.

Use of the *Anatomic Region Sequence* data element or *Primary Anatomic Structure Sequence* data element assumes that the body part is categorized either as part of the imaging order or at the time the image is obtained. Unfortunately, discrete anatomic information of any kind is not routinely populated today even in many radiologic imaging studies. For cross-sectional modalities such as CT, MR, and PET, in particular, vendors have been slow to provide any structured anatomical information in any dedicated data element, and users have had to depend on free-text data elements like *Study Description* (0008,1030) and *Series Description* (0008,103E). Historically, the values in *Body Part Examined* have proven to be remarkably unreliable, perhaps because downstream usage of the anatomic information has not justified applying discipline earlier in the workflow or adding features to the acquisition devices [29].

These deficiencies in implementation and deployment will need to be addressed if the *Anatomic Region Sequence* is to be used to drive image consumption workflows in enterprise imaging. The relative novelty of most visible light imaging applications in terms of both implementation and deployment provides an opportunity to get this information right from the start, by correctly populating the *Anatomic Region Sequence*. This process is particularly straightforward for dedicated devices that can only image a single region (for example, a nasolaryngoscope). However, more general capture devices, such as a smartphone camera, require a user interface and a source of relevant codes.

### Study Description (0008,1030) and Series Description (0008,103E)

Typically in radiology and cardiology, these plain text data elements are populated automatically by the acquisition modality from text passed from the order through the DICOM modality worklist, or the data element is configured in the operator-selected acquisition protocol. In encounter-based workflows in which there is no order, the capture device may have contextual information from the EHR to populate the *Study Description* or *Series Description* in a meaningful manner. In the worst case, this value is entered by the operator.

*Study Description* has traditionally been used to drive the selection of comparison studies during image interpretation workflows within a single facility. However, it has severe limitations. *Study Description* may describe many attributes of the acquisition process that are not related to anatomy nor relevant to the retrieval decision, its values are not selected from a standard ontology, and each institution can define how the data element will be populated. The lack of standardization of the *Study Description* values limits the comparison of data across disparate organizations. Even within a single facility, there may be considerable variation [30]. There have been many attempts to standardize radiology procedure codes for ordering, and the meanings of such codes may be useful for populating *Study Description* in a consistent manner [31–35]. Unfortunately, this harmonization has been largely unsuccessful.

*Series Description* is more often used to drive display protocols than to select comparison studies. However, it too is subject to considerable variability, particularly for complex acquisitions such as MRI where series names may vary by vendor, modality, protocol, and operator [36, 37].

On a global scale, since both *Study Description* and *Series Description* contain plain text values, they are likely to be localized, both with respect to language and character set. Accordingly, even though international image sharing or telemedicine may not be the primary driver for image metadata interoperability, interoperability is a significant factor in the generalizability of tools marketed multi-nationally. These many deficiencies make *Study Description* and *Series Description* undesirable choices to drive enterprise imaging workflow going forward. These values may be needed as a last resort when integrating legacy devices and archived images. The preferred coded attributes, like Anatomic region Sequence, are populated with values from a standard ontology and will help to enable international image sharing. Ideally, standard ontologies include synonyms for concepts in multiple languages while the concept itself, which is language-independent, is uniquely identified using a standard code value.

### Institutional Department Name (0008,1040), Institutional Department Type Code Sequence (0008,1041)

The *Institutional Department Name* describes in text form the department housing the equipment that acquired the images. Traditionally, it is intended for fixed equipment that does not move, like most CT and MR scanners, and is usually configured and relatively static in the device itself. However, for portable and mobile devices, it would ideally contain information about the modality operator’s department at the time of acquisition. For example, in the framing use case, the images from the nasolaryngoscopy would ideally be labeled with “otolaryngology” in the *Institutional Department Name*. This label would be straightforward if the device was owned and maintained by the otolaryngology department. However, if the device was shared by multiple services and used exclusively in the emergency department, the correct *Institutional Department Name* value would be more difficult to populate. Additionally, there is no standard set of text strings to unify the representation of different departments across different facilities, or even within the same institution. The DICOM standard provides a coded equivalent, *Institutional Department Type Code Sequence* (0008,1041). However, this field is rarely used in practice. A standard set of codes is defined for Institutional Departments, Units, and Services to use in the *Institutional Department Type Code Sequence* data element [38]. However, many specialties are not yet included in this code set while other specialties have multiple subspecialties represented. For example, Wound Care is not included in the code set, yet 13 different codes are defined for Radiology. Additionally, patient-submitted photographs cannot be labeled using this code set.

A challenge of identifying studies using the departmental location of the device is that many disease processes are cared for by providers from multiple specialty divisions. Most specialties use other forms of imaging than their own, especially images obtained by the radiology, cardiology, and pathology departments. Information about the device department may be less relevant than information about who ordered the imaging procedure. Despite this limitation, *Institutional Department Name* and *Institutional Department Type Code Sequence* values may be helpful for creating display protocols.

In summary, it is recommended that for enterprise imaging applications, *Institutional Department Type Code Sequence* (0008,1041) should be populated with an appropriate code and that receiving systems be prepared to accept absent values in the *Institutional Department Name* element.

### Reason for the Requested Procedure (0040,100A), Reason for Performed Procedure Code Sequence (0040,1012), Admitting Diagnoses Description (0008,1080), Admitting Diagnoses Code Sequence (0008,1084)

In the setting of enterprise imaging, medical images can be obtained for multiple purposes including screening, evaluation of specific signs or symptoms, confirmation of a diagnosis, documentation of a clinical finding, or continued assessment of a known disease process. Even though some application’s worklist, management, and reporting services have mechanisms for conveying diagnosis, many systems do not have this capability. Additionally, systems that do have the capability to display a diagnosis do not have the capability of displaying the information from each diagnosis-based data element. For example, the free-text field, *Admitting Diagnoses Description* (0008,1080), and its preferred coded alternative, *Admitting Diagnoses Code Sequence* (0008,1084), may be used to encode a diagnosis that is already known before the imaging is performed. The DICOM standard does not define which of the many standard coding schemes should be selected, since there are typically local, regional, and national requirements that dictate the choice.

When an imaging procedure is requested, there may be a coded *Reason for the Requested Procedure* (0040,100A), which may be copied from the worklist into an item of *Request Attributes Sequence* (0040,0275), and if the reason remains applicable to what was performed, copied into *Reason for Performed Procedure Code Sequence* (0040,1012). Alternatively, the latter could be selected by an operator from an appropriate list of values on the acquisition device or application. When there is no order, such as in encounter-based workflow, contextual information taken from the encounter or manually entered codes may be available with which to populate *Reason For Performed Procedure Code Sequence* (0040,1012). Again, DICOM does not define a standard code set. Whatever choice has been made in the EHR for coding reasons for procedures can be propagated through the worklist or configured in the acquisition device.

Although it seems straightforward in our use case to assume that all of the image sets were obtained in the setting of nasopharyngeal carcinoma, closer consideration reveals that many of the images were obtained before the diagnosis of nasopharyngeal carcinoma was confirmed. When the initial images were obtained, the family medicine physician had a differential diagnosis that included nasal polyps and nasopharyngeal carcinoma among several other entities. The reasons for the imaging included a “mass” and “bleeding” or more specifically, “epistaxis”, and there was, as of yet, no admitting diagnosis. Once the MRI was obtained, a presumptive diagnosis of nasopharyngeal carcinoma was established, but this might still not have been elevated to the level of a diagnosis in the EHR. The diagnosis was not confirmed until the histopathology had been finalized.

It is important to remember that DICOM images and their embedded metadata are usually not updated as more information becomes known. The DICOM data elements recorded in images are designed to include values that are known at the time of acquisition. Accordingly, when a diagnosis is made after the initial disease presentation, it is usually not possible to go back and relabel all the imaging studies that were, in retrospect, related to the specific disease process. That said, there is value in populating both *Reason For Performed Procedure Code Sequence* (0040,1012) and *Admitting Diagnoses Code Sequence* (0008,1084) for enterprise imaging use cases if the appropriate information is available.

### Problem: Interoperability

As enterprise imaging grows, it is crucial that data can flow between systems and be understood by both the originating and receiving applications [39]. As in other areas of medicine, it is important to consider interoperability within an organization and between two or more disparate organizations. We will consider both examples below.

### Interoperability Within an Organization

An early tenet of enterprise imaging proposed that specialists care for their patients at a specialty level while simultaneously sharing the acquired images and their associated metadata to all care providers. Although this concept is discussed in relation to the tools needed in an image acquisition device or an image viewer, it can also be applied to metadata. As we share images between different entities, it is essential that metadata be standardized as an important vehicle for a common vocabulary. For example, an otolaryngologist may require very specific metadata for images obtained during nasolaryngoscopy, particularly when a biopsy is obtained. In our use case, it may be important for the otolaryngologist to identify the location of the mass as originating from the opening of the Eustachian tube, the torus tubarius, or the fossa of Rosenmüller. Similarly, for the gross pathology photograph obtained after lymph node dissection, it may be important for the pathologist to identify the resected cervical lymph nodes as arising from level II or level III. Even though this level of specificity is important for otolaryngology and pathology-based images, it is less important for identifying radiologic images when the entire head and neck is imaged.

This example highlights one of the fundamental challenges that must be accounted for when selecting a body part terminology. Namely, the terminology must be an ontology including anatomic relationships, so that concepts with a higher degree of specificity are relatable to those with a lower degree of specificity. In this example, the terms Eustachian tube, torus tubarius, or fossa of Rosenmüller would all be child terms of the less specific anatomic structure, the nasopharynx. Additionally, the cervical lymph node levels II and III would be child terms of the less specific anatomic structure, cervical lymph nodes. Finally, the terms nasopharynx and cervical lymph nodes would themselves by child terms of the even less specific anatomic structure head and neck. An ontology with this type of structure would allow specialists in one field to select the appropriate level of granularity while allowing the specialists in other fields to consume the data in more general categories.

### Interoperability Between Two (or More) Disparate Organizations

Patients often move between providers practicing at different organizations. This situation is particularly true as patients see providers across different specialties. In order to realize the promise of enterprise imaging, the images obtained by one provider must be consumable by any provider at any organization. Standards facilitate seamless data transfer and consumption.

As enterprise imaging evolves, we believe that it is likely that different practice groups will make different decisions regarding the level of specificity needed in the *Anatomic Region Sequence* data element. In our use case, the first otolaryngologist works in a private, office-based practice but the second otolaryngologist works in an academic hospital-based practice. It is possible that the first otolaryngologist always uses the term nasopharynx for the *Anatomic Region Sequence* for every image obtained during nasolaryngoscopy. This otolaryngologist uses this data element for simplicity and efficiency purposes. Because the second otolaryngologist works in a teaching hospital and would like to use the body part data element to produce trainee teaching files or support research, she populates the *Anatomic Region Sequence* field with the specific location of the primary abnormality, the fossa of Rosenmüller.

From an enterprise imaging perspective, it is impossible to anticipate all possible uses of the stored images, and hence to pre-select an appropriate level of granularity or a hypothetical small subset that is the lowest common denominator that all senders and recipients could agree upon. Such a simplistic approach is regarded as a dead-end that will likely satisfy no one. Accordingly, we do not believe it is appropriate to routinely transform a less specific term to a more specific term. Given additional information, it may be desirable and possible to morph a data element value to a more specific data element when sharing images with other facilities, but the complexity and limited return on investment may not justify the work effort or the risk of an error. The obvious solution is the same as for intra-institutional deployment: when data is transferred, the sending and receiving systems should be able to understand the ontological relationships of the concepts coded in the *Anatomic Region Sequence* data element.

### Problem: Privacy

As visible light photographs are added to enterprise imaging archives, there will be heightened interest in patient privacy and data security. Although these issues exist today, the influx of a large number of sensitive photographs will highlight many issues. Patients will ask for clarification and demand a higher level of privacy and security related to images of sensitive body regions or uniquely identifiable body parts such as the face. Although the issues related to the technical details of managing access to sensitive photographs are beyond the scope of this white paper, it is apparent that the Anatomic Region Sequence data element can play a role in enabling access control measures in the EHR and imaging archives.

In our use case, there are several instances when potentially sensitive photographs were acquired. If clinical photographs of the entire face were obtained, these images are considered to represent protected health information [40]. During the patient’s routine mammography visit, a photograph of her breast was acquired. Although this information was recorded to assist the breast imaging specialist, patients may want additional restrictions like “break the glass” (access granted on demand but flagged for increased security audit scrutiny) placed on access to this image relative to the restrictions normally applied to the X-ray mammography images.

In these examples, the *Anatomic Region Sequence* data element can be used to identify sensitive images quickly. For example, any photographs labeled with a body part related to the external genitalia, the breast or the whole face could automatically be marked as sensitive, and access restrictions or a higher level of auditing applied. It is important to note that *Anatomic Region Sequence* data element is not the only data element likely to be useful for identifying sensitive photographs, nor that all sensitive photographs can be identified in an automated manner using this data element. However, the anatomic information, already acquired for other purposes, may be the easiest to implement and maintain until more sophisticated attribute-based or role-based approaches are deployed.

## Challenges to Address

Although we believe that the *Anatomic Region Sequence* data element is a crucial data element required to implement enterprise imaging successfully, there are several challenges that need to be addressed. In the next section, we will identify many of these and describe the complexity that any body part ontology standard should resolve.

### Challenge: Multiple Body Parts

We anticipate that providers will frequently take photographs of multiple body parts, just as radiographers image multiple body parts. There are at least four different scenarios to address when considering this challenge: one photograph containing multiple contiguous body parts, one photograph containing multiple noncontiguous body parts, multiple photographs containing contiguous body parts, and multiple photographs containing noncontiguous body parts.

### One Photograph Containing Multiple Contiguous Body Parts

An example of this scenario is a patient who falls into a glass window causing a laceration extending from the palm of the hand, across the wrist, to the proximal forearm near the elbow. Before placing sutures, the Emergency Medicine provider may take a single photograph of the laceration.

This scenario is perhaps the most straightforward. In this example, most providers would select a single term that best identifies the image. However, any selected anatomy-based ontology must be able to account for this naming structure and have a term for the selected contiguous body parts. Here, the term lower arm would be more precise than the term forearm. The major challenge with this approach is identifying the correct terms for groupings of contiguous body parts, especially as different groupings of body parts are added. A standard that provides a hierarchical relationship among contiguous body parts would be desirable to meet this need.

### One Photograph Containing Multiple Noncontiguous Body Parts

There are two major variants of this scenario, potentially with different solutions.

In the one scenario, a provider may take a single photograph of paired structures or those that possess cross-midline symmetry, such as both hands. This may be done for side-by-side comparison of normal and abnormal or where a systemic disease affects both sides. In this case, an appropriate coded value to use in *Anatomic Region Sequence* data element may be “hand”. The multiple body part challenge is traditionally addressed by adding a laterality modifier such as right, left, or bilateral. Additionally, a single pre-coordinated code (such as “both hands”) could be used.

In the second variant, two different noncontiguous unpaired body parts, such as the forearm and the leg, are photographed at the same time. For example, an emergency medicine nurse may take this type of photograph to show how a single traumatic event, such as a gunshot, affected both structures. In this case, there may be no appropriate single coded term to use in the *Anatomic Region Sequence* data element. It would not be practical to predefine every possible combination and permutation. The practical solution is to use the least general single coded term that includes both parts, such as extremity. However, we anticipate that in many cases, the most general term (i.e., whole body) will be used. The use of the more general terms such as whole body fails to distinguish between situations when parts of the specified structure are imaged as opposed images of the entire specified structure. For example, there will be scenarios in which the entire body is included in a single photograph.

### Multiple Photographs Containing Contiguous Body Parts

In this example, one abnormality may affect multiple body parts. For example, a large vascular malformation may extend from the upper arm through the hand. A provider caring for this patient may take photographs of each structure.

One approach to this scenario is to take advantage of the distinction between the regional description encoded in *Anatomic Region Sequence*, and the more detailed location that may be encoded in the *Primary Anatomic Structure Sequence*. Specifically, for the entire set of photographs acquired together to document the malformation, a single code for *Anatomic Region Sequence* could be used, such as “upper limb”. Even though *Anatomic Region Sequence* is an image-level data element, it can repeat for all images in the same DICOM Series or Study. For each image, a more specific value, like “hand”, could be used in *Primary Anatomic Structure Sequence* to provide the more granular information that is known to the creator.

To achieve this, the system would ideally automatically select the appropriate parent term that contains all the individual child terms representing the body parts photographed. The major challenge with this potential solution is the same as identified for the single photograph with multiple contiguous body parts scenario, namely, the need to use concepts drawn from a system with a robust ontology that defines the structural relationships between body parts. For example, in this vascular malformation use case, the most appropriate *Anatomic Region Sequence* data element for the study would be the “upper limb”. There are also limits to such hierarchies and ontologies though. For example, if the vascular malformation extended to the chest wall, there might not be a single pre-coordinated concept that for the combination of the lateral part of the chest and one arm [41].

### Multiple Photographs Containing Noncontiguous Body Parts

An example of this scenario occurs when a patient has a rash that affects multiple body parts such as the face, back, and hands. To document the rash, the provider may take photographs of each body part. Similar situations occur in radiology, such as performing skeletal surveys for suspected child abuse, metastatic disease, or rheumatoid arthritis.

If the acquisition device or user takes no specific action to document the combination, then each image can and should have the appropriate single value in *Anatomic Region Sequence*, which is an image-level data element. Specifically, the face images will be labeled “face” and the hand images labeled “hand”. This simplistic approach is not only consistent with the normal pattern of use but also facilitates re-use. For example, when hand images are needed as relevant images, regardless of in what context they were acquired.

This is true regardless of how the acquired images happen to be grouped into DICOM studies or different series within a study. Institutional policy may be to create a different study for each anatomic region to allow for an anatomy-specific text value in *Study Description*, or the converse, to group all the images acquired in a single context together and use a generic, or combined, plain text *Study Description* (e.g., “rash survey” or “face, back, and hands”). If *Anatomic Region Sequence* is correctly populated at the image level, the ability of the end-users to consume the images may not be compromised.

We recommended not using a single combined value for *Anatomic Region Sequence* and depend on more specific *Primary Anatomic Structure Sequence* values for this scenario since it unnecessarily complicates the consumer’s task, for which clear communication of the distinct body parts is likely to be more relevant than the fact that the images were acquired as a combination.

### Challenge: Internal Structures

Many of the same challenges described in the multiple body parts section apply to the internal organs. We will highlight several of these examples and identify new body part challenges.

### Specificity of Labeling Internal Structures

The use case and the discussion related to data consumption and the differing level of specificity needed among specialists highlighted this challenge. Another example could be used in gastroenterology. An upper endoscopy, pill endoscopy, flexible sigmoidoscopy, and colonoscopy are all different procedures, each imaging the gastrointestinal tract. However, each creates photographs and videos of different segments of the gastrointestinal tract. Many of the challenges in this example are similar to the challenges described in the scenario of multiple photographs containing contiguous body parts. However, it is important to again recognize that the specific terms relating to the gastrointestinal tract must not only map to the parent term of the gastrointestinal tract but also the larger body part structures such as chest for the esophagus, and abdomen for the stomach, small intestines, and colon. This type of mapping will allow for appropriate comparison with other imaging studies, such as CT and MRI enterography.

In this section, we will briefly address the relationship between *Anatomic Region Sequence* and *Primary Anatomic Structure Code Sequence* as it pertains to the labeling of internal structures in the DICOM image header.

### Multiple Internal Structures

Sometimes, a surgical photograph will include multiple internal structures. The challenge in this scenario is similar to the challenges identified in the noncontiguous body part examples. Like those examples, this may represent a single photograph or multiple photographs. Interestingly, these two examples may be handled differently. For example, a photograph or video of open abdominal surgery may contain the liver, spleen, and different segments of the gastrointestinal tract. In this scenario, even though each organ is disparate and in other cases would be considered to be non-contiguous, when all of the organs are displayed together, the photograph may be optimally classified as a single structure: the abdomen. However, if each organ is imaged individually in the same patient, there may be a need to label each structure individually, similar to the multiple photographs including noncontiguous body parts scenario.

### Specimens

Gross specimen images, photomicrographs of single fields as well as digital whole slide images are described somewhat differently than other DICOM images in that there is a specific Specimen Module that is used to both identify and describe specimens [42]. Theoretically, any DICOM image may be of a specimen and use this module; however, it was intended mainly for pathology. Anatomy information for the specimen is encoded using *Primary Anatomic Structure Code Sequence*, nested within items of the *Specimen Description Sequence* (0040,0560) [43].

In particular, *Anatomic Region Sequence* is not present in the Whole Slide Microscopy Information Object Definition (IOD), and *Primary Anatomic Structure Code* Sequence is not present in the top-level data set, but rather is nested as described above [43].

Histopathology specimens present other challenges. In most cases, structures can be labeled according to the organ of origin or sub-part of it (e.g., “left upper lobe of the lung”). However, there will be scenarios where this does not apply. In patients with diseases such as leukemia or Langerhans cell histiocytosis, it may be more important to label the type of cell that is imaged. Pathologists should have the same flexibility to label at the specificity needed for their work as other specialists. Just as for other specialties for which this increased specificity is needed, anatomical concepts should be drawn from an ontology that describes the appropriate relationships between organs, cellular structures, and disease states.

This extends to the need to identify sub-cellular structures. Some of the most widely used examples include karyotypes and electron microscopy. These photographs are of structures in each cell or many cells within the body. It is important to use a sufficiently rich anatomic ontology that includes the relevant structures.

There are instances in which multiple body parts are resected simultaneously. For example, in a multi-visceral transplant, the liver, pancreas, and bowel are transplanted en bloc. A photograph of the donor organs would have many of the same challenges as described in the multiple internal structures section.

When a biopsy is obtained, only a tiny fraction of the structure is removed. For example, while a liver biopsy only represents 1/50,000 the volume of the liver [44], the specimen should be labeled “liver.” This also applies to specimens of body fluids such as blood, urine, and cerebral spinal fluid, which are included in most anatomical ontologies.

### Challenge: Non-anatomic Structures

Non-anatomic structures are uniquely challenging. We anticipate that as enterprise imaging expands, providers will photograph non-anatomic structures, such as the bloody packing material described in the use case. Throughout the following discussion, care should be taken not to undermine the semantic intent of the standards and data elements used in the name of expediency.

In many of the following scenarios, the root cause of tension in the model is the desire to associate an imaging subject that is not the patient with the patient’s record and their other images. Encoding such images as part of the patient’s imaging record is expedient, but other than for phantom and quality control images, there is no specific way in DICOM to signal that these are of a different subject. Additionally, the concept of “anatomy” may or may not be relevant. For example, with regard to the photograph of nasal packing material, the material comes from the nasal cavity and thus could be labeled similarly to the other images of the patient’s cancer. Although this may work for structures related to a specific anatomic structure, it does not work for all photographs. For example, a photograph of an automobile after a motor vehicle collision has relevance to a patient’s injury, but it does not have associated anatomic relevance.

One potential solution to this problem is to use the *Device Sequence* (0050,0010) [45]. Though primarily intended to describe devices visible in human images, it could have applications in the context of the more general discussion in this section.

### Mixed Body Fluids and Substrate Material

Although the use case presents a straightforward example of packing material containing blood, the potential data element could be different depending on the contents. For example, if the packing material contained a mixture of dried blood and pus, the choice of the label becomes more difficult. Although the anatomic location of the packing material could be used, the composition of the body fluid may be more important than the originating structure. One approach would be to encode “wound packing material” in *Device Sequence* and “nose” in *Anatomic Region Sequence*. This would ensure that these images would be selected as relevant when looking for images of the “nose.”

### Tumors

The majority of tumors should be labeled based on the organ or structure of origin. There are some tumors where this may be difficult. Examples of this include large infiltrative tumors, multifocal metastatic tumors where the organ of origin is unknown, and large soft tissue tumors that span multiple body parts. In some instances, the same rules that apply to other photographs of multiple body parts can be applied. Other instances, where the organ of origin is unknown, are more challenging. In this case, we recommend that the image is labeled with the most appropriate *Anatomic Region Sequence*. For example, a large, infiltrative abdominal tumor could be labeled as “abdomen.”

### Implants/Foreign Bodies

Implants and foreign bodies represent extreme examples of the same issue: a non-biologic structure has been placed into the body. In the broadest sense, the difference between the two represents the intentionality of placement of the foreign structure. We can envision photographs being obtained of the foreign item regardless of placement intent.

There are unique challenges to address. For example, before performing a hip replacement, an orthopedic surgeon may take a photograph of the hip implant or a cardiologist may take a photograph of a pacemaker before the device is implanted. In these examples, the *Anatomic Region Sequence* should represent the placement location for the device. Although this principle seems straightforward, the complexity increases with lines, drains, and small implantable devices like vascular coils. We believe that in this example, organizations will likely decide which *Anatomic Region Sequence* coded value best applies for each line, tube, or small implanted device. As described above, the use of *Device Sequence* to describe the device, and *Anatomic Region Sequence* to describe its actual or intended location may be a sensible pattern to follow.

Foreign bodies could be labeled based on the body part of origin or body part location. For example, a splinter lodged in the sole of the foot may be labeled as a foot even after the splinter has been removed. Foreign bodies that span multiple body parts, such as bullet fragments, may be more difficult. However, we believe that the resolution could be similar to the labeling solution selected for the scenario of a single image containing multiple noncontiguous body parts.

### Other Living Entities

Other living entities will be photographed as providers care for patients. This may be more important in locales where parasites or insects, such as ticks or bed bugs, are more common. These organisms may have been identified by the care provider during the care of the patient or may have been photographed or killed and brought into the hospital by the patients. In either case, photographs of the organisms are important to help document the patient’s condition and to help identify the potential pathogen, venom, or irritant related to the organism.

We propose two possible solutions to this problem. First, the organ of origin could be used to guide body part labeling. Although this labeling may be appropriate in some instances, it may not be appropriate in others. For example, lice could be labeled with the body part of the head or scalp and the *Ascaris lumbricoides* parasite could be labeled as the gastrointestinal tract. However, there will be some cases where the other living entity affects multiple body parts (bed bugs) or affects an unknown body part (venom in the bloodstream, but no visible bite). It is also important to remember that plants may be included in this category and may cause similar effects when touched or ingested.

This is one category that does not fit well into the approach used for devices.

### Other Non-living Entities

Similar to implants/foreign bodies, other non-living entities may be photographed to be included as a part of a patient’s medical condition. For example, a paramedic may photograph illicit drugs after caring for a patient who has overdosed or a parent may take a photograph of an ingested liquid that their child has swallowed. The issues related to these photographs are similar to those identified in the previous two sections. In general, the *Device Sequence* data element can be used to describe the non-living entity, and the *Anatomic Region Sequence* data element can be populated with the affected organ or site of injury.

### Challenge: Annotation of Image Context

Though beyond the scope of this paper, we note in passing that the same structured coded approach is applicable to annotation of regions of interest with in an image. Such annotations are typically encoded in separate objects rather than the images themselves. The annotation objects (e.g., DICOM Structured Reports (SR) and Segmentations (SEG)) include mechanisms for using the same structured codes as are used in *Anatomic Region Sequence*.

## The Path Forward

Regardless of whether the future classification and labeling of images for any specialty is performed by a human operator or a machine, there will remain a need for standardizing the mechanism for communication of the label [46].

In this white paper, we have described why we believe that the Anatomic Region Sequence data element is the one where standardization is most urgently needed. Using a nasopharyngeal carcinoma framing use case, we have demonstrated the importance of this data element in data consumption, interoperability and data privacy. In addition, we have tried to identify challenges that need to be addressed for a body part ontology to be accepted and account for many of the use cases in enterprise imaging.

We believe that the next step is for providers and industry representatives to work together to identify and select an existing body part ontology that will meet the varied needs of specialists and the general end-users across the enterprise. Once this group proposes a standard ontology, vendors must be encouraged to support this standard to enable the promise of enterprise imaging. Additionally, a new or extended IHE Integration Profile for acquisition workflow specifically addressing anatomic information may be needed.
